# Recommended Best Practices for Lyophilization Validation—2021 Part I: Process Design and Modeling

**DOI:** 10.1208/s12249-021-02086-8

**Published:** 2021-08-18

**Authors:** Feroz Jameel, Alina Alexeenko, Akhilesh Bhambhani, Gregory Sacha, Tong Zhu, Serguei Tchessalov, Lokesh Kumar, Puneet Sharma, Ehab Moussa, Lavanya Iyer, Rui Fang, Jayasree Srinivasan, Ted Tharp, Joseph Azzarella, Petr Kazarin, Mehfouz Jalal

**Affiliations:** 1 Abbvie, North Chicago, IL USA; 2 Birck Nanotechnology Center, Purdue University, 1205 W State St., West Lafayette, IN 47907 USA; 3 Merck, Kenilworth, NJ USA; 4 Baxter Healthcare, Bloomington, IN USA; 5 Pfizer, Andover, MA USA; 6 Genentech, South San Francisco, CA USA; 7 BMS, New Brunswick, NJ USA; 8 Fresenius Kabi, Buffalo, NY USA; 9 Ultragenyx pharmaceutical Inc., Brisbane, CA USA

**Keywords:** freeze-drying, lyophilization, process design, process optimization, controlled ice nucleation technology (CIN)

## Abstract

**Abstract:**

This work describes lyophilization process validation and consists of two parts. Part I focuses on the process design and is described in the current paper, while part II is devoted to process qualification and continued process verification. The intent of these articles is to provide readers with recent updates on lyophilization validation in the light of community-based combined opinion on the process and reflect the industrial prospective. In this paper, the design space approach for process design is described in details, and examples from practice are provided. The approach shows the relationship between the process inputs; it is based on first principles and gives a thorough scientific understanding of process and product. The lyophilization process modeling and scale-up are also presented showing the impact of facility, equipment, and vial heat transfer coefficient. The case studies demonstrating the effect of batch sizes, fill volume, and dose strength to show the importance of modeling as well as the effect of controlled nucleation on product resistance are discussed.

**Graphical abstract:**

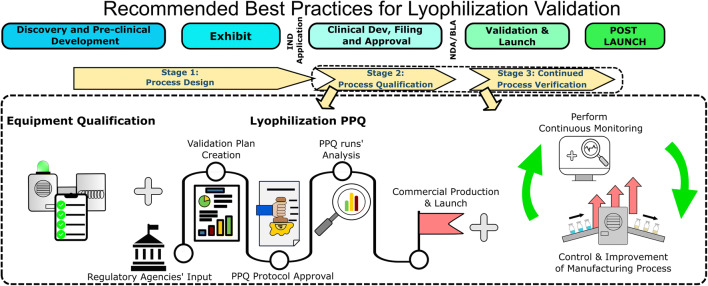

## INTRODUCTION

Pharmaceutical product stability can often be improved by removing water or other solvents in a controlled manner through the process referred to as lyophilization or freeze-drying (1). Lyophilization serves as one of the most widely used techniques for manufacturing solid biopharmaceuticals, including but not limited to biologics (2) and vaccines (3), to achieve the intended shelf-life of the product during storage and shipping. Such improvement in stability enhancement is attributed to limited hydrolytic reactions coupled with restricted mobility and/or conformational flexibility of the active molecule in presence of excipients. The pharmaceutical lyophilization involves three main steps (4): (1) freezing of the product which is initially in a solution to produce a matrix of ice and other crystallizable excipients while concentrating other solutes and the active pharmaceutical ingredient (API) within the interstitial voids; (2) primary drying, wherein ice is sublimed at low temperature, vacuum conditions; (3) secondary drying to remove unfrozen water, which may be adsorbed on the surface of the crystalline phase or is in the solute phase, carried out at temperatures well above that in the primary drying. The equipment and the processes are designed to ensure product sterility is maintained during the process of lyophilization. Furthermore, during the early stages of product development (pre-pivotal studies), there is a great emphasis on process design space as it allows for process understanding, process monitoring, and product characterization while establishing a rational line of sight to commercial manufacturing.

The lyophilization process design, therefore, is a critical aspect of manufacturing process development for a lyophilized pharmaceutical product. A well-understood process can be scaled up and controlled, resulting in consistent quality attributes across product batches, which can be demonstrated by validation. Commercial product launch requires that the lyophilization process is successfully validated per country-specific regulatory expectations. This is especially important when there are changes to the product or process such as different dosage strengths or lyophilizer equipment. A critical understanding of the factors affecting the product quality associated with a lyophilization process can enable such changes to be carried out using fewer engineering runs. This can often be further supported by leveraging models to predict heat and mass transfer in various scenarios.

This work is the first of a two-part paper describing the current state of lyophilization validation. Part I will focus on process design, while part II will discuss process qualification and continued process verification. Also, both articles will provide the authors’ perspectives on best practices for lyophilization validation as well as the use of modeling to support comprehensive and efficient validation. The intent of these articles is to provide readers with recent updates on lyophilization validation, supplementing past publications by Jennings in 1986 (5) and Trappler in 2007 (6). Ever since the publication of the latter report, several advances have been attained in lyophilization technologies, process analytical technology (PAT), computer modeling, and simulation tools for lyophilization process and equipment capability. Accordingly, an update of the best practices of the validation of lyophilization processes is needed especially given the surge in the number of therapeutic modalities in development pipelines that require lyophilization. This work is our community-combined opinion and industrial prospective on the lyophilization validation process.

Part I of this best practices publication focuses on the early lyophilization process design with an emphasis on the generation of a design space for a given product and equipment combination. In addition, strategies for engineering runs during commercial scale-up are proposed, including considerations for lyophilization cycle design and optimization, and equipment capability. The benefits of modeling as applied to process design and scale-up are also discussed and illustrated through case studies, addressing challenges such as multiple vial sizes, fill volumes, and dosage strengths. Product formulation and container closure systems, while related to lyophilization, are described in limited detail to the extent that they are relevant to process design and scale-up. New and upcoming approaches to process improvement (controlled ice nucleation or CIN for example), product monitoring, and process understanding (tunable diode laser absorption spectroscopy or TDLAS and process analytical technology or PAT for example) are also listed below with an emphasis on chemistry, manufacturing, and controls (CMC) requirements associated with manufacturing a safe, effective, and quality products.

## LYOPHILIZATION PROCESS VALIDATION

Process validation is generally defined as “the collection, documentation, and evaluation of data from the early development stages through commercial production to establish a manufacturing process that is capable of consistently delivering a quality product” (7). In this regard, process validation involves a series of product and process development activities as well as manufacturing operations and is classified into three main stages: process design, process qualification, and continued process verification. As part of the validation process, the drug product to be lyophilized must be well-defined and documented for its physical, chemical, and pharmaceutical properties, and all aspects of finished product such as moisture, sterility, dose uniformity, stability, etc. must be captured. Best practices and guidance on process design are captured below with an emphasis on the unit operation of freeze-drying only.

### Stage 1—Process Design

The main goals of the process design stage are (1) to build and capture process knowledge and understanding and (2) to establish a strategy for process control (7). The commercial manufacturing process is defined during this stage based on knowledge gained through developmental experiments and scale-up activities. The process design experiments do not need to be performed under good manufacturing practice (GMP) conditions but must be based on scientifically sound methods and should be adequately documented and verified (7).

Building and capturing process knowledge and understanding requires that all relevant data obtained during drug product and process development activities are collected and documented. At this stage, process design should consider the functionality and limitations of commercial manufacturing equipment due to geometry and design, scale effects, the variability of component lots, environmental conditions, and measurement systems. Typically, risk analysis tools such as failure modes and effects analysis (FMEA) or cause and effect analysis can be used to evaluate critical variables that can potentially impact process performance and product quality and to guide the design of the studies required to build sufficient process understanding (8). It should be noted that any change in either the equipment, facility, process itself, or even the test method should be well-evaluated to identify and document the rationale and/or need for revalidation or requalification. Computational models and simulations based on first principles can also help the design of the experimental studies by establishing the relevant process parameter ranges to be tested and, in many cases, may eliminate the need for a design of experiments (DoE) based on statistical approach thereby simplifying the change management process.

Based on the process knowledge and understanding obtained from laboratory and pilot-scale experiments, a strategy for process control is established to ensure the consistency of product quality, typically by reducing and/or adjusting for input variation during manufacturing. In the lyophilization process design, a design space diagram is usually constructed to determine the safe operating zone for critical process parameters. Process control typically involves monitoring critical equipment and process parameters and may involve process analytical technologies (PAT) to enable adjusting the processing conditions to maintain critical parameters within target limits. The control strategy, in general, may include process parameters and critical quality attributes (CQAs) related to the drug product, raw materials, and excipients, facility, operating conditions of the lyophilize, in-process checks (IPC), product specification along with testing approach, and frequency of monitoring and control (Annex I). Closed-loop control of primary drying for process acceleration and utilization of Control Ice Nucleation (CIN), for example, has been a key area of discussion the community to attain faster and uniform drying and minimize/eliminate failure during scale-up process. These emerging topics and their impact on process design would also be discussed in subsequent sections below.

### Stage 2—Process Qualification

The goal of the process qualification (PQ) stage is to determine if the process designed in stage 1 is reproducible for commercial manufacture and as such activities in this stage should apply cGMP-compliant procedures. Stage 2 involves qualification of the facility, equipment, and utilities as well as process performance qualification (PPQ). Further details on stage 2 as applied to lyophilization validation are well documented in part II of the best practices paper. It should be noted, however, that the goal of process validation here is to demonstrate that the lyophilization process leads to the desired product characteristics and quality under all load conditions (i.e., bracketing the minimum and maximum load) and thus a few different case studies demonstrating the power of simple modeling to accommodate process and product changes are highlighted in the “Power of Simple Modeling for Process Optimization and Scale-up” section.

### Stage 3—Continued Process Verification

The goal of stage 3 is to ensure that the process remains in a state of control; that is, it consistently assures the continued process performance and product quality (7). Thus, as part of continued verification, process monitoring data are needed to evaluate if the drying process performs as documented, including the performance of CPPs (critical process parameters) within tolerance limit and thus the value of in-process analytics (e.g., moisture, potency) and in-line PAT (e.g., TDLAS) cannot be overstated. Further details on continued process verification for pharmaceutical lyophilization are given in part 2 of this best practices paper.

## STAGE 1—PROCESS DESIGN

### Generation and Use of Design Space

#### Introduction to the Driving Forces and Resistances During Primary Drying

The primary drying step in a lyophilization process is conducted to remove bulk ice from the frozen solution. This is accomplished by tuning shelf temperature and chamber pressure to achieve sublimation while controlling the product temperature. The product temperature is critical during the process, but it cannot be controlled directly. It should be noted that pre-lyo formulations are characterized by their glass transition (T_g’_), eutectic temperature (T_eu_), and collapse temperature (T_c_); the product temperature is monitored using a thermocouple; however, the drying operation itself is a time/temperature/pressure-driven process. It is desirable to operate at a product temperature as high as possible without causing failure of the product. The failure is defined as the loss of structural integrity of the drying solid that often results from exceeding a critical product temperature. Thus, properties of the final formulated product such as T_c_/T_eu_ are rendered critical and are well-characterized before starting the drying process. Operating at a product temperature that is as high as possible is desired because the driving force during primary drying is the difference in the vapor pressure of ice between the sublimation front and the chamber pressure. The temperature in the condenser is typically less than approximately −60 °C and the temperature at the sublimation front is typically much higher. For example, the vapor pressure of ice at −60 °C is approximately 8.1 mTorr, and the vapor pressure of ice at the sublimation front at a temperature of −20 °C is approximately 774.4 mTorr. The large pressure difference establishes a flow of water vapor from the area of high vapor pressure to the area of low vapor pressure making it advantageous to perform at the highest product temperature possible, creating the most efficient process. Resistance to heat and mass transfer in this dynamic process renders controlling product temperature constant as drying progresses a challenging task. This is further described briefly below.

The resistance to heat transfer originates from the materials through which the heat must travel to reach the product. These materials include the fluid flowing through the shelves, the stainless steel shelves, the primary packaging in contact with the shelves, and the air space between the bottom of the primary packaging and the shelves. The heat transfer coefficient of the primary container (K_v_) differs from container to container and is dependent on the chamber pressure. The heat transfer coefficient represents the ratio of the heat flow from shelves to the product in a given vial, the outer cross-sectional area of the vial, and the temperature difference between the shelf surface and the product at the bottom of the vial (9). Therefore, it is important to measure the K_v_ for the specific primary packaging container and chamber pressure; if the type and/or manufacturer of the packaging changes, K_v_ needs to be measured again. Additionally, since the free molecular heat conductivity changes with pressure, the value of the apparent K_v_ also changes as a function of pressure (9).

There is also resistance to the mass transfer of water vapor through the drying product (R_p_). The water vapor must travel from the sublimation front via the pores of the dried layer and through the gap between the container and container closure to reach the chamber. The factors that affect R_p_ are the degree of supercooling before ice nucleation, the physical nature of the solids, the solids content, and the location of the sublimation front within the drying solid (10). For example, solutions that undergo a high degree of supercooling will reach low product temperatures before ice nucleation. It should be noted that product resistance can be affected by annealing conditions and fill height (11). The ice nucleation temperature affects the time available for the crystallization of ice. Solutions that have less time for ice crystal growth will have small pores in the drying solid and this increases the R_p_. Similarly, high concentrations of solids will also have narrow pores through which water vapor must travel. Finally, R_p_ is lowest at the onset of drying and increases as the sublimation front travels to the bottom of the solid during drying. The R_p_ value ultimately used for drying solids is often based on the worst-case scenario when the sublimation front reaches the bottom of the drying solid.

#### Equations for the First Principles of Heat and Mass Transfer

Data for the K_v_ of the vials and R_p_ of the product can be collected during the cycle and used in heat and mass transfer equations to create a design space graph for primary drying. K_v_ is calculated using the following system of ordinary differential equations:

1 $$ \frac{dq}{dt}={K}_v{A}_v\left({T}_s-{T}_b\right) $$

2 $$ \frac{dq}{dt}=\Delta  {H}_s\frac{dm}{dt} $$

where $\frac{dq}{dt}$ is the heat transfer rate in Joule·h^−1^, K_v_ is the vial heat transfer coefficient in Joule·h^−1^·cm^−2^·°C^−1^, A_v_ is the outer area of the vial in cm^2^, T_s_ is the temperature of the shelf surface in °C, T_b_ is the temperature of the product in contact with the bottom of the vial in °C, $\frac{dm}{dt}$ is the mass flow rate in g·h^−1^, and ∆*H*_*s*_ is the heat of sublimation of ice in Joule·g^−1^ (9). The equations are rearranged based on the data that are available to the following:

3 $$ \Delta  {H}_s\frac{dm}{dt}={K}_v{A}_v\left({T}_s-{T}_b\right) $$

4 $$ {K}_v=\frac{\Delta  {H}_s\frac{dm}{dt}}{A_v\left({T}_s-{T}_b\right)} $$

The vial heat transfer coefficient K_v_ changes as a function of pressure due to the significant influence of gas conduction at the typical pressures encountered in pharmaceutical freeze-drying (12). K_v_ is often characterized using a tray of the specific vial or other primary packaging container filled approximately half full of water. The water is frozen and a vacuum is initiated as the shelf temperature is adjusted to the shelf temperature planned for use with the product. As shown in Eq. (4), the mass flow rate (or rate of ice loss), shelf, and product temperatures are needed to calculate K_v_.

**Fig. 1:**
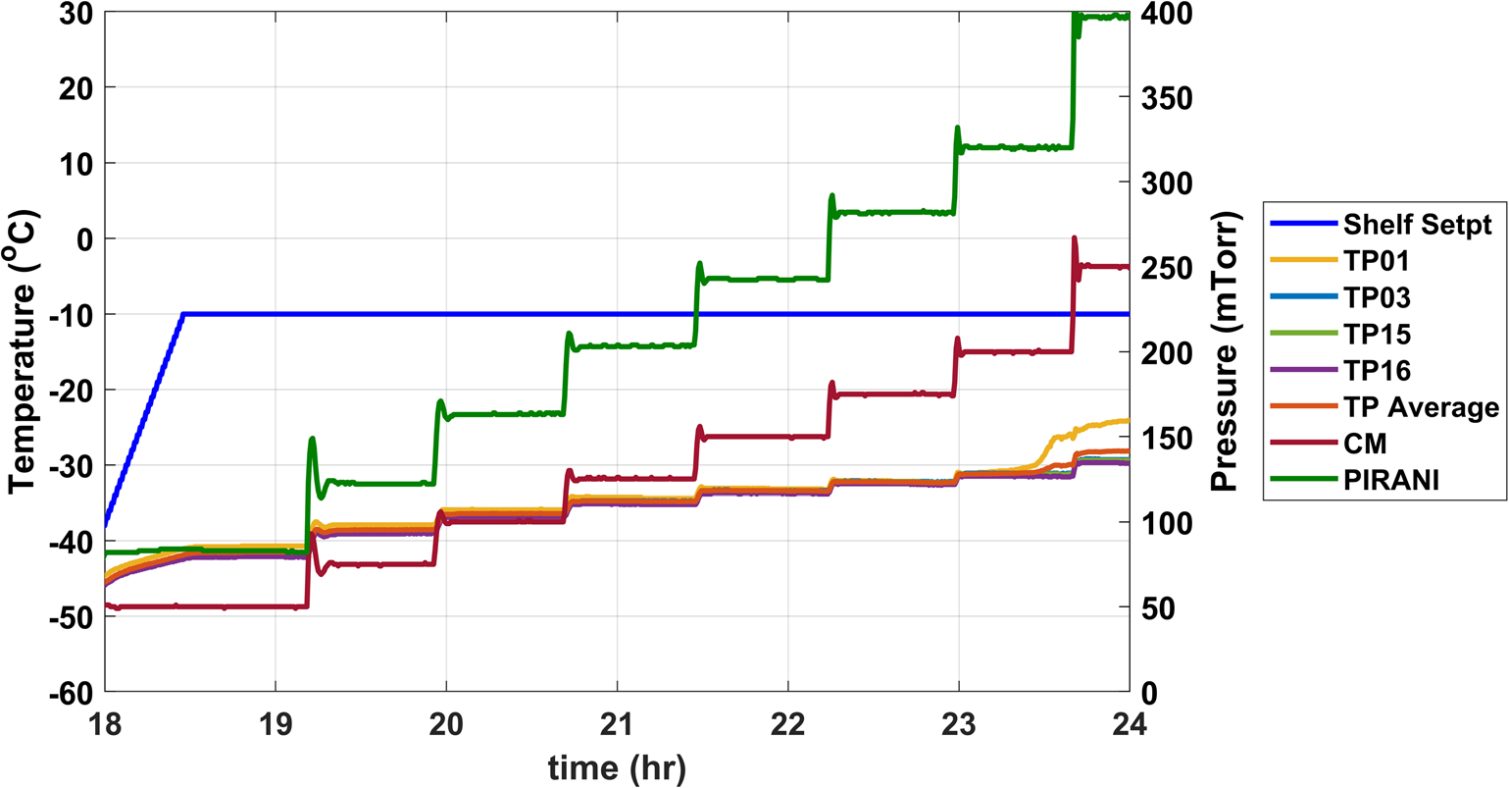
Representative plot of process parameters during K_v_ measurement. TP refers to product temperature probes numbered 1, 3, 15, and 16. CM refers to the capacitance manometer reading. PIRANI refers to the Pirani Gauge reading

The rate of ice loss is calculated either gravimetrically or by measuring the in-process mass flow of water vapor at multiple increments of chamber pressure using tunable diode laser absorption spectroscopy (TDLAS) (13). For example, a tray of vials can be equipped with type-T thermocouples that are placed in vials located at the center, front, and back of the tray. TDLAS is a mass flow meter that is located in the duct that connects the product chamber with the condenser. The instrument uses 2 lasers and 2 detectors to measure the concentration and flow rate of water vapor traveling to the condenser. The data are used in the first principles of heat and mass transfer equations to calculate Kv and R_p_ (14). Referring back to the tray of vials as per the example, the tray is transferred to the shelf of a lyophilizer and the vials are frozen to −45 °C. When using TDLAS, an operational check is performed for zero-velocity offset and then primary drying is conducted at a shelf temperature specific to the product. The chamber pressure is set at 50 mTorr and the sublimation rate is monitored by TDLAS. The shelf is held at the set chamber pressure until a steady state is reached. The chamber pressure setpoint is increased to 75 mTorr, 100 mTorr, 125 mTorr, 150 mTorr, 175 mTorr, and 200 mTorr, allowing sublimation to reach an equilibrium at each setpoint. A representative plot of the process data is provided in Figure 1.

Approaches to obtaining information on K_v_ vary across industry. Some approaches use a batch average value for K_v_ (e.g., by using TDLAS) to develop a design space while other methods determine the K_v_ based on the location of the container on the shelf and between shelves by relying on gravimetric approaches. The end goal with these characterizations is to understand what influences K_v_ and to use a consistent approach for the development of the design space. Irrespective of the approach used, an analysis of Eq. (4) reveals that K_v_ can be obtained by measuring the normalized mass flow rate and the temperature differential between product (T_b_) and shelf (T_s_). To measure T_b_, it is recommended that the thermocouples should be placed at the bottom of the vial. As the sublimation front approaches the bottom, the measured temperature closely approaches the temperature of the sublimation front. The vapor pressure, P_i_, is then calculated by using a least-squares fit of the data. This yields an equation relating vapor pressure and product temperature in the form (15):

5 $$ {P}_i=\mathit{\exp}\ \left[\frac{A}{T_b}+B\right] $$

where T_b_ is the temperature at the bottom of the frozen layer and the values of constants A and B vary based on the range of temperatures being used. For temperatures between 169 K and 273.16 K, which are typical for lyophilization processes, *A* =  − 6132.9 and *B* = 28.868 when P_i_ is measured in Pa (15,16).

**Fig. 2:**
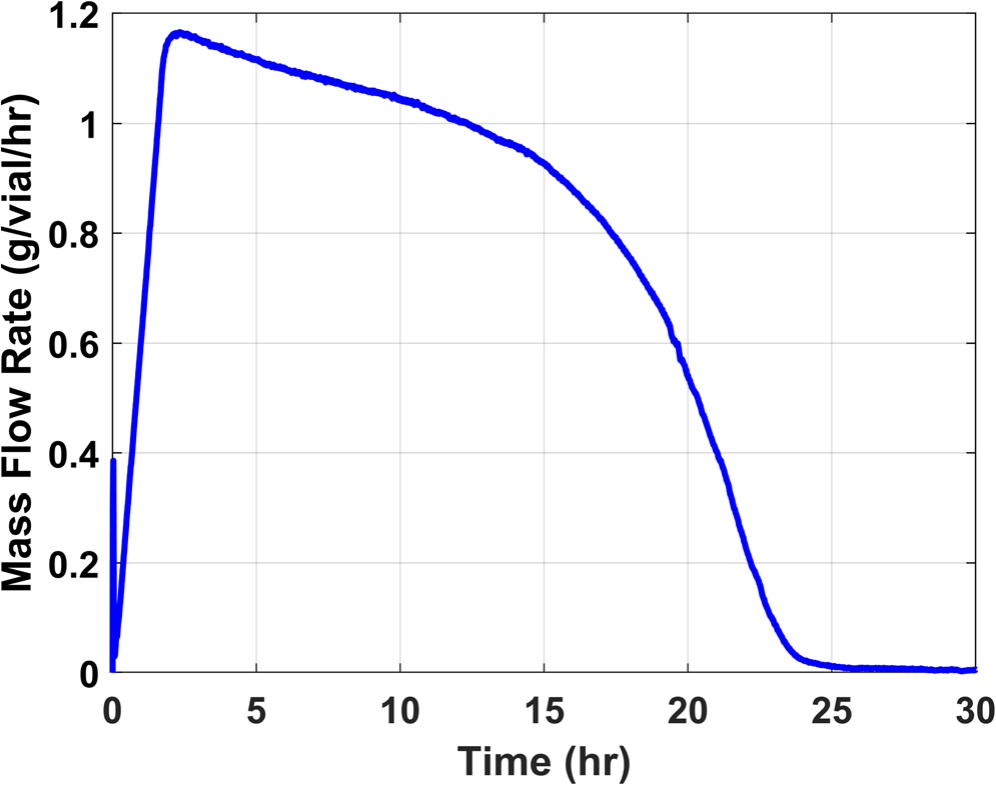
Graph of mass flow rate of water vapor as a function of time for the batch

Similarly, the mass flow rate can be either obtained gravimetrically or using TDLAS as an in-line PAT tool. It should be noted that TDLAS is a non-invasive tool that allows concurrent measurement of the mass flow rate during the freeze-dry cycle. An illustrative example of a TDLAS profile observed during a freeze-drying cycle wherein the batch average mass flow rate is recorded as a function of time is shown in Figure 2.

**Fig. 3:**
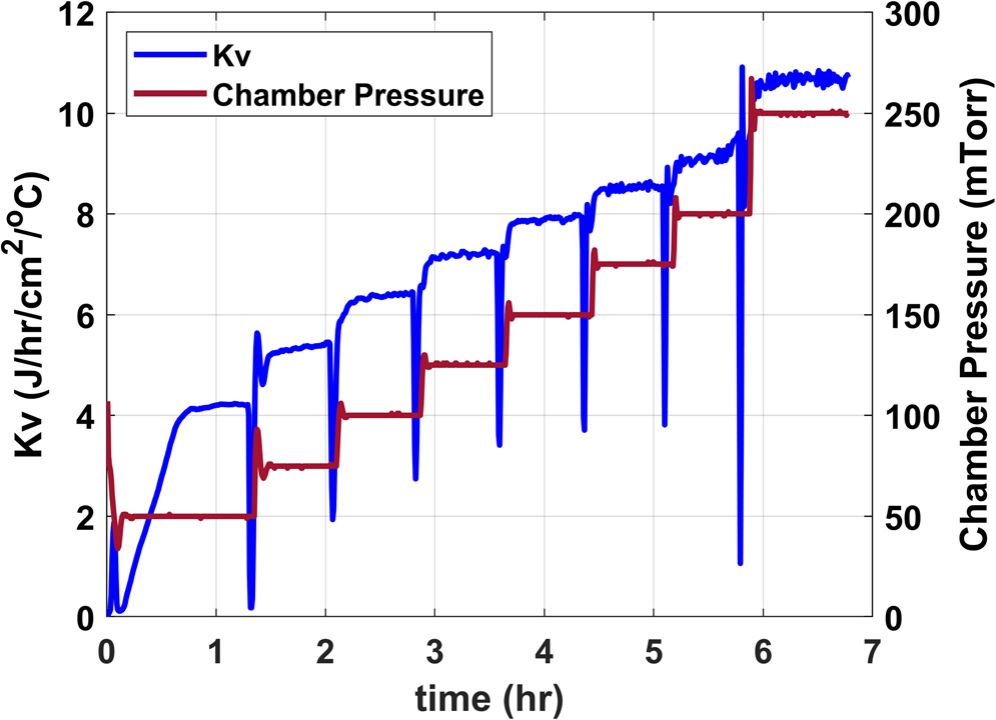
Representative plot of K_v_ as a function of chamber pressure and time

Using the mass flow data obtained from the TDLAS, the shelf temperature (T_s_), and product temperature (T_b_), K_v_ is calculated for each chamber pressure in Joule·hr-1·cm-2·°C. A representative plot of K_v_ as a function of chamber pressure is provided in Figure 3.

Furthermore, as observed in Figure 2, the mass flow rate decreases from about 5 h through 15 h before a large decrease is observed. The sublimation rate decreases steadily during primary drying because the resistance to vapor flow increases with the depth of the partially dried layer and thus R_p_.

**Fig. 4:**
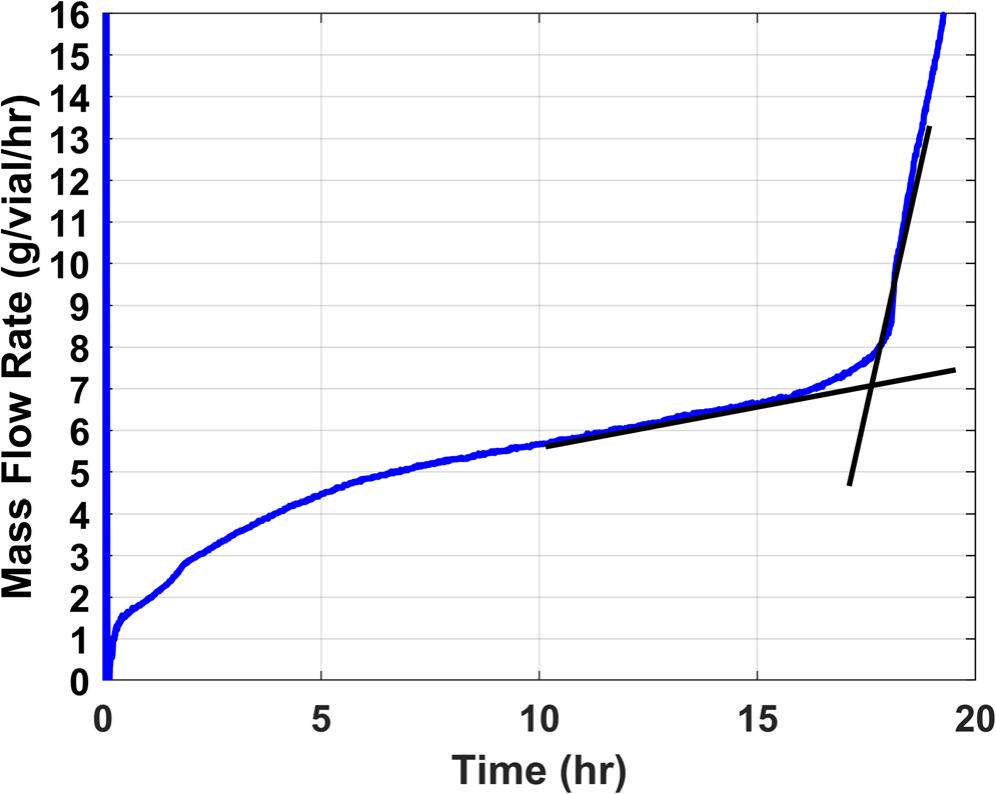
R_p_ as a function of time with the highest resistance observed at approximately 17 h showing an R_p_ of 7 Torr·hr·cm^2^·g^−1^

The product dried cake resistance, R_p_, in contrast to K_v_, is assumed to be independent of shelf temperature and chamber pressure and is a function of formulation and drying characteristics. R_p_, given in cm^2^·Torr·h·g^−1^, is calculated using the following equation:

6 $$ {R}_p=\frac{A_p\left({P}_i-{P}_c\right)}{\frac{dm}{dt}} $$

where A_p_ is the cross-sectional area of the product (using the inner diameter of the vial), P_i_ is the vapor pressure of ice at the sublimation front, and P_c_ is the chamber pressure. Using the mass flow rate, the partial pressure of ice, and partial pressure of water vapor in the chamber, the resistance is calculated in Torr·hr·cm^2^·g^−1^. R_p_ increases with time during primary drying with the maximum value of R_p_ observed towards the end of primary drying (Figure 4). There is a sharp increase in R_p_ directly after 17 h of primary drying time. This occurs after all ice sublimes and resistance becomes a function of diffusion of unfrozen water through the drying solid. Therefore, the point of highest resistance before complete loss of ice is considered the point of the curve just before the rapid increase in R_p_.

R_p_ and K_v_ are combined with the critical product temperature for the product and the equipment capability curve to create a design space graph. The calculations are entered into an Excel® macro (or equivalent software) to solve the model equations and calculate the product temperature at different combinations of shelf temperature and chamber pressure. The calculations can also be conducted iteratively to create the design space graph. In general, multiple methods can be used to complete the calculations for a design space as long as they are developed based on the main equations for R_p_ and K_v_ presented above (17). Some lyophilizers are equipped with software that allows for the determination of R_p_ and K_v_ in tandem and some equipment determines R_p_ and K_v_ using manometric temperature measurement (18). It is worth noting that the design space generated for lab-scale equipment cannot be directly applied to commercial-scale lyophilizer since the scale-up procedure needs to be performed with the parameters (i.e., K_v_, R_p_, minimum controllable pressure) adjusted accordingly (19).

#### Determination of Primary Drying conditions and Construction of Design Space

The relationship between the process inputs, such as chamber pressure, shelf temperature, and the critical quality attributes, can be described within a design space. The International Council for Harmonization of Registration of Technical Requirements for Pharmaceuticals for Human Use (ICH) guidance Q8 (R2) defines “design space” as “the multidimensional combination and interaction of input variables (e.g. material attributes) and process parameters that have been demonstrated to assure quality.” Working within the design space is not considered a change. Movement out of the design space is considered to be a change and would normally initiate a regulatory post approval change process. Design space is proposed by the applicant and is subject to regulatory assessment and approval. The reader is reminded that as part of continuous process verification, controls and run charts from historical data allows for monitoring any atypical patterns/trends in process parameters and any quality attributes over time and thus assures the manufacturing process is in a state of control during the product lifecycle.

There are multiple approaches used for defining the appropriate conditions for primary drying. An empirical approach is to select the critical product temperature regardless of the use of thermal characterization. The process is developed using a target product temperature, shelf temperature, and chamber pressure that provide acceptable appearance (no loss of structural integrity), residual moisture, and reconstitution characteristics as well as a stable and sterile product, at a laboratory-scale equipment. Subsequent cycles are conducted using shelf temperatures ±5 °C from the original shelf temperature set point and ±20 mTorr around the original chamber pressure set point to verify product and process robustness/tolerance. The space for the operation would then be within the tested shelf temperatures and chamber pressures. The challenge with this approach is that the point of failure for the product may not be known and the behavior of the formulation at low temperature may not be known. Additionally, such an approach results in a non-optimized process thereby impacting the operational efficiency of the facility. These data are often crucial to understanding the physical behavior of the formulation and to developing a process with sufficient data to support possible future excursions during manufacturing.

A related approach is to use a statistical design of experiments. The experiments are designed by varying the processing factors such as shelf temperature and chamber pressure within a specified range. The experiments are conducted and the effect of the factors on drying time, product appearance, and stability is examined. Using statistics in this manner does not necessarily account for the combined influence of shelf temperature and chamber pressure on product temperature. Like in the first case, it is completely feasible to select different levels of shelf temperature and chamber pressure without even affecting the product temperature. This approach can provide a false sense of security if the influence of the process parameters on product temperature is not fully understood. Besides its lack of product and process understanding, this approach also suffers from non-optimal process parameters thereby reducing the operational efficiency of the facility.

Both approaches described above, however, can result in a freeze-drying process that is completely acceptable. The challenge is that the studies may not be based on a thorough scientific understanding of process and product.

An improved approach that is continually being refined is the development of a primary drying design space. The design space described below is based on first principles and includes all relevant data needed to understand the product, process, and their interaction. The design space is created using data on the capability of the equipment, the K_v_ for the specific vial, the R_p_ for the formulation, and the critical product temperature (to characterize failure modes) for the product. The critical product temperature is defined through thermal analysis and failure point studies during primary drying. The two methods identify the product temperature at which failure occurs and the conditions at which they occur. It is good practice to set the target product temperature a few degrees below the critical product temperature to ensure the product temperature of the vials located on the edges of the shelves does not approach the failure point.

The product resistance R_p_ and heat transfer coefficient K_v_ define the governing relationship between shelf temperature, chamber pressure, and product temperature needed to achieve the maximum sublimation rate without compromising the product quality. These data are used to calculate the combinations of shelf temperature and chamber pressure that ensures that the product temperature remains below the defined critical product temperature.

The use of the design space depends on knowing the vapor removal capability of the lyophilizer. The equipment capability is defined as the maximum sublimation rate (kg/h) for a given chamber pressure. The maximum sublimation rate at a given chamber pressure corresponds to the equipment limitation and in many cases represents the choked flow conditions for lyophilizers designed with a spool piece between the product chamber and the condenser (20). The choked flow occurs when the flow of water vapor leaving the chamber reaches the speed of sound, and flow within the spool piece is the rate-limiting factor of water vapor reaching the condenser (21). If a sublimation rate exceeds the vapor removal capability of a lyophilizer at a given chamber pressure, a build-up of vapor will occur with the product chamber pressure increasing over the setpoint, reaching minimum controllable pressure P_min_. The value of P_min_ as a function of the sublimation rate is often determined using ice slabs on all shelves of the lyophilizer using the lowest pressure possible for the lyophilizer. The shelf temperature is increased incrementally with pauses in between to determine equilibrium pressure at a given sublimation rate (22,23). The challenge is that such ice slab experiments are often difficult to perform once the lyophilizer is used for routine production.

**Fig. 5:**
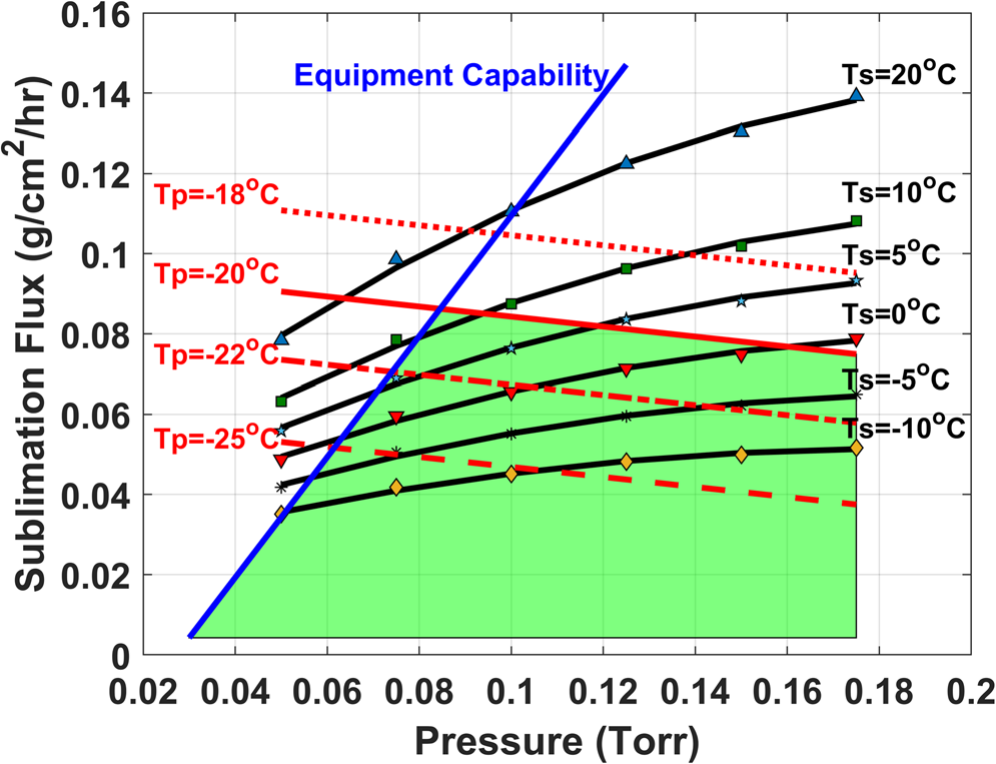
Example of a primary drying design space graph showing sublimation rates as a function of pressure and temperature. Green area is the safe zone of operation. The red traces are the calculated product temperature isotherms. The solid red trace is the experimental critical product temperature. The black traces are the calculated shelf temperature isotherms. The equipment capability is represented by the solid blue line

There are different methods for creating a primary drying design space. One method is to construct a graph plotting the sublimation rate as a function of chamber pressure and temperature (Figure 5). This method provides the conditions for the most efficient process and all of the conditions that ensure that product temperature remains below the critical product temperature, but the graph does not include how processing time is affected by the conditions. Processing time may also increase when transferring the process to an aseptic environment where higher degrees of supercooling can be expected due to the clean environment.

A conservative approach is used to create the design space by decreasing the critical product temperature to account for the warmer temperatures experienced by edge vials. This should prevent encountering product failure if the process conditions align with the equipment capability and/or critical product temperature borders. It is good practice to challenge the borders of the design space to determine the likelihood of product failure, choked flow of the equipment, and to examine the effect of the processing conditions on primary drying time.

The design space featured in Figure 5 does not include primary drying time within the graph. It depends on knowing the effect of the process conditions on the primary drying time. This data can be obtained by testing the boundaries of the design space which also confirms the applicability of the design. Primary drying time may be longer when the process is transferred to full-scale manufacturing. This is typically tested using a demonstration batch to confirm the cycle.

**Fig. 6:**
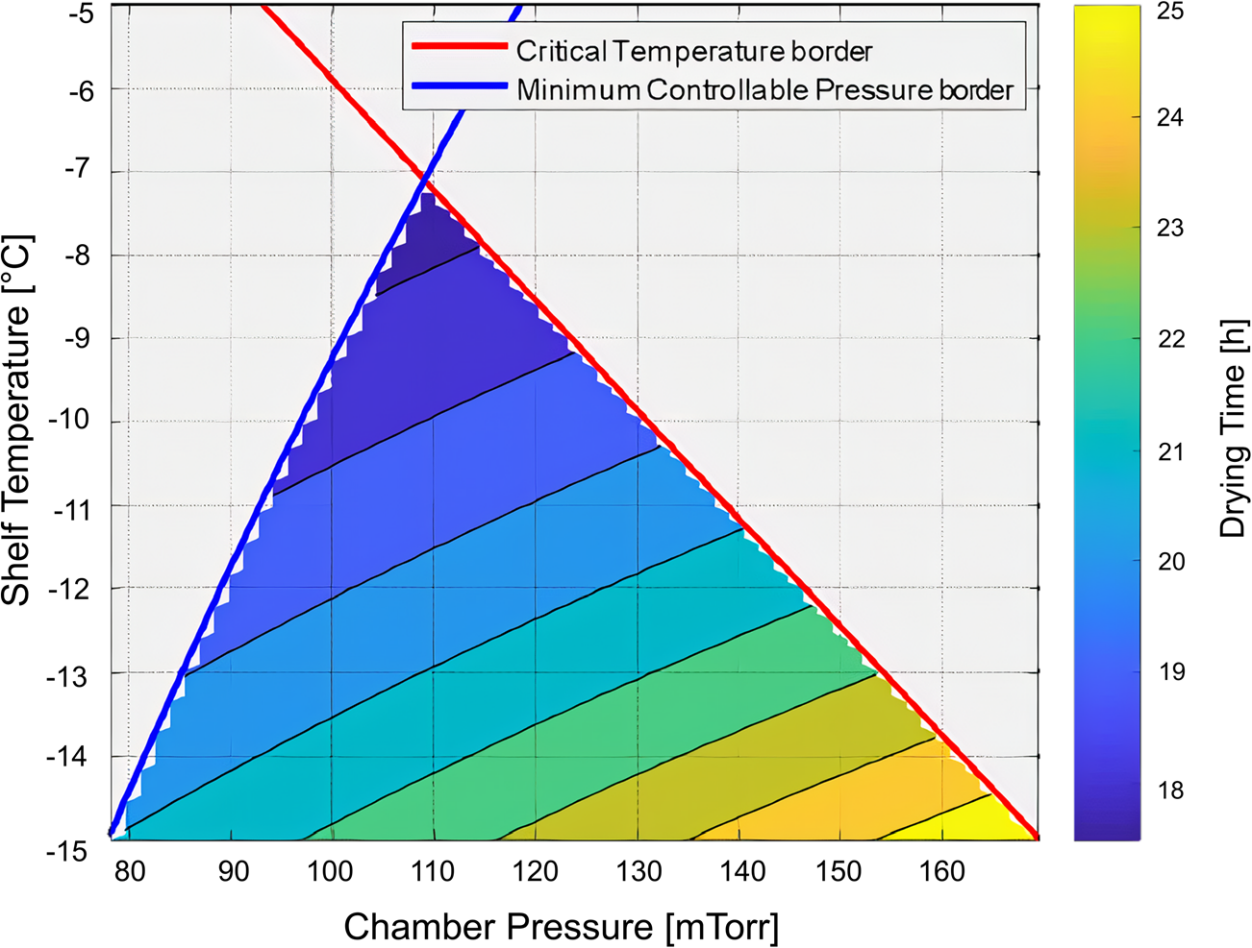
Graph for primary drying design space that incorporates time as a function of chamber pressure and shelf temperature. The area under the critical temperature border and minimum controllable pressure border shows the safe zone of operation where the drying time increases with higher shelf temperature

Fundamentally the same information presented in Figure 5 can also be visualized using time within the graph (Figure 6). Incorporating time as a function of shelf temperature and chamber pressure allows for the prediction of total primary drying time for particular product within the safe zone of operation. The safe zone of operation is between the borders for critical temperature and the minimum controllable pressure (choke point).

Both graphs featured in Figures 5 and 6 are flexible about changing vial types, even from different manufacturers, if needed. A change in vial requires only the determination of the K_v_ for the vial and incorporating the data in the existing graph if the fill volume does not drastically change as R_p_ is a function of fill volume.

The first design space graph in Figure 5 assumes the worst-case R_p_ for the drying solid which occurs near the end of drying when ice is at the bottom of the drying solid. The second design space graph simulates the process for each process parameter combination. The latter approach provides the changes in R_p_ with respect to the location of the sublimation front in the drying solid. R_p_ will be lowest at the start of drying and increases as the sublimation front lowers in the drying solid. This suggests that shelf temperature and chamber pressure can be adjusted throughout the process based on the R_p_. For example, more aggressive conditions can be used at the beginning of the process when R_p_ is low. Also, different processing conditions within the design space can result in different R_p_ values for some formulations. Particularly, R_p_ can be affected by only freezing conditions during a lyo process (24). Primary drying should not have any impact on R_p_ unless there is any loss in structure due to collapse or meltback. The change in R_p_ within the safe zone suggests that it is good practice to test the boundaries of the design space to confirm that the physical properties of the solids are acceptable.

### Engineering/Development Runs at Commercial Scale

It is a standard practice within the industry to perform commercial-scale runs testing the lyophilization process before moving forward with process performance qualification (PPQ) runs. At scale, runs are not a cGMP requirement but are completed to minimize risk before proceeding to PPQ. Depending on the company, these runs may be referred to as engineering, development, or demonstration runs, but in all cases, the lyophilization process, along with other unit operations in the formulation, filling, and inspection, is being tested to identify any unexpected changes that might occur during the transfer from small-scale runs or in tech transfer to a new site.

Several different product filling strategies have been embraced by the industry for the completion of engineering runs. If possible, a surrogate or a placebo, formulations without the API, is used during development to minimize API requirements. A placebo is the drug product formulation without any API, typically excipients and water for injection. The removal of the API can lead to different drying behavior for the remaining solution, and therefore may not be fully representative of the drug product formulation. In a surrogate, the API is replaced with a material substitute, such as human serum albumin or Dextran-60 for a therapeutic protein, in order to provide similar solution concentrations and thermal behavior for the lyophilization process. Another option to minimize API requirements while collecting data on the active drug product is to first fill the lyophilizer with a surrogate and then replace surrogate vials with active vials at all locations where analytical testing would be performed. The number of engineering runs to be completed can vary based upon knowledge of the product formulation, lyophilization process, and equipment being used. Among the LyoHub (Advanced Lyophilization Technology Hub, (25)) member companies, however, a single successful engineering run is the goal of technical transfers for a given dose. If there are multiple-dose presentations for the same formulation, the engineering run strategy may be further minimized using a bracketing approach to reduce the amount of formulated drug product used within the studies.

The engineering run can provide a wealth of information as part of the process evaluation and should be completed in a manner as close to the PPQ runs as possible while allowing appropriate time for analysis of data generated during the run. Goals from the run should be to confirm that product temperature performance is within the acceptable limit, to perform a visual inspection of vials by location, and to determine the total primary drying time at scale. A sampling of the run generally follows the five locations per shelf, which are the front, back, center, left, and right sides of the lyophilizer, with a focus on appearance, residual moisture, reconstitution time, and any other product-specific attributes of interest. Visual inspection of the lot is also completed to assure uniform cake appearance for the batch. Based upon the evaluation of the engineering run data, a decision to move forward with the PPQ batches for a product is made.

## POWER OF SIMPLE MODELING FOR PROCESS OPTIMIZATION AND SCALE-UP

There are several benefits of applying modeling described in the “Generation and Use of Design Space” section to the lyophilization process that helps both industry and the patient besides gaining a better understanding of the process. By applying and implementing the modeling, one can reduce the number of experiments during the development that would free up resources and material requirements and in limited cases may also result in a reduction in the cost of goods manufactured (COGM). By reducing the number of experiments, the development time can be reduced enabling faster availability of medicine to patients with life-threatening diseases. Additionally, modeling helps to better understand and design a robust process enabling the availability of a safe and high-quality drug to the patients.

The lyophilization processes can be modeled based on the fundamental understanding of heat and mass transfer given in the “Generation and Use of Design Space” section. At steady state, when heat input is equal to output, the heat transfer rate due to shelf heating and sublimation cooling rate can be equated and the unknowns can be determined using the following equation (26):

7 $$ \left[\ {T}_{pr},\frac{dm}{dt},{t}_{PriDry}\ \right]=f\left(\ {T}_{sh},{P}_{ch},{K}_v\left({P}_{ch}\right),{A}_v,{l}_{pr,0},{R}_p,{c}_{solid}\right) $$

where $$ {l}_{pr,0}=\frac{V_{fill}}{A_p} $$

### Development and Optimization of a Lyophilization Process

**Fig. 7:**
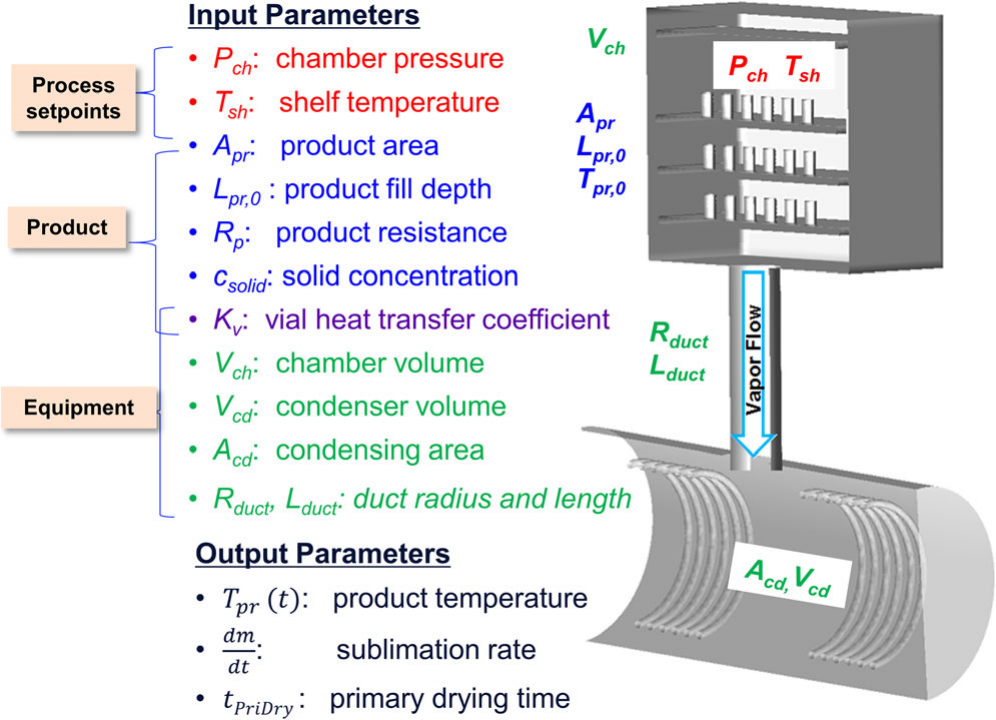
Lyophilization model inputs and outputs

Historically, a lyophilization process is developed by trial and error methods where, for a given collapse temperature of a formulation, experiments are performed with various shelf temperature and chamber pressure values until the output parameters product temperature and product quality at the end of freezing and drying phases match with the target product temperature and product quality attributes. This requires several experiments to be run and consumes a lot of resources, time, and material. However, with the use/application of modeling, one can use key inputs to estimate output process parameters as outlined in Figure 7. These parameters must be obtained on a specific lyophilizer for the target product so that the model based on these inputs are representative of the actual lyophilization process at that specific lyophilizer.

**Fig. 8:**
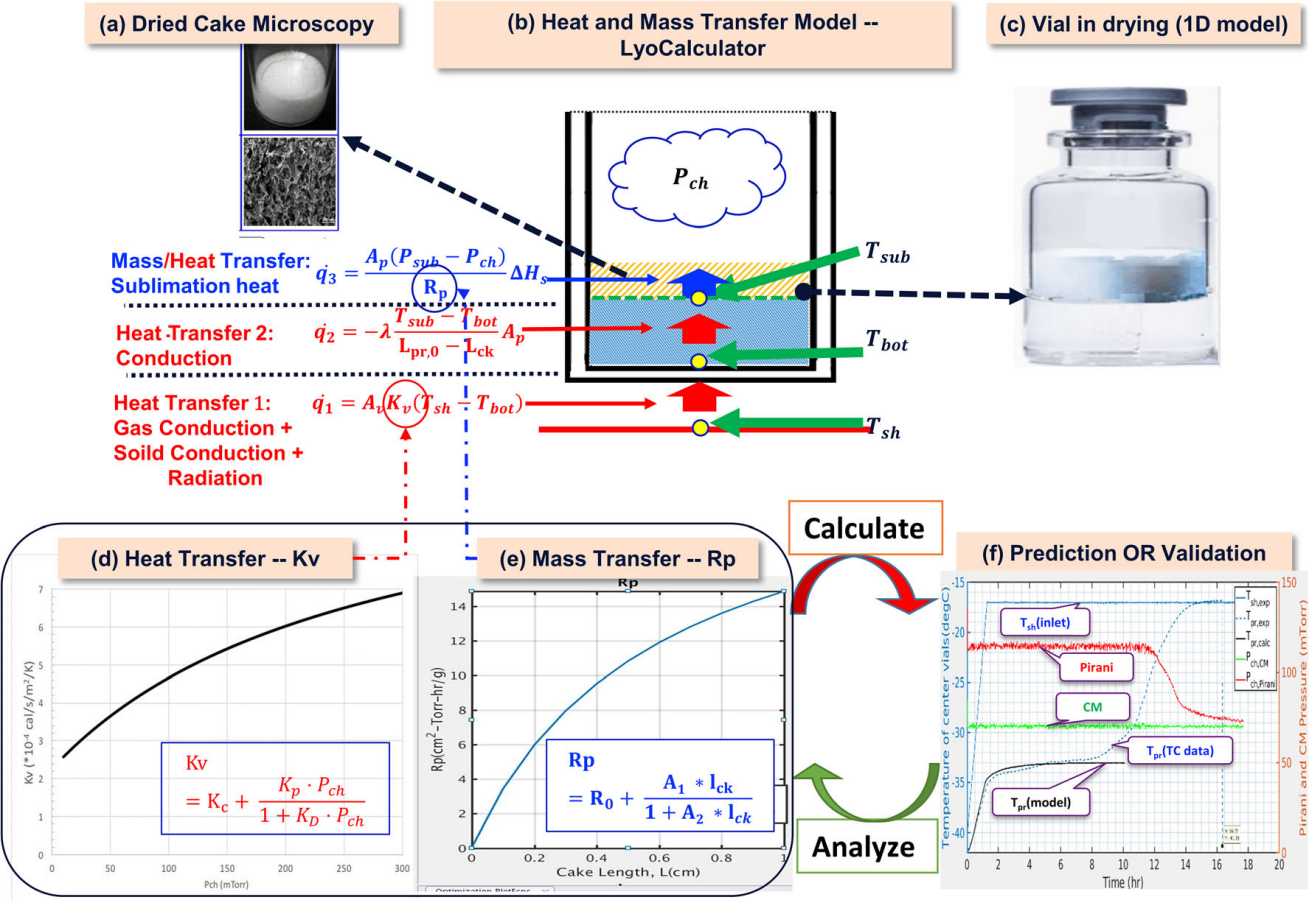
Heat and mass transfer modeling equations and the application in lyophilization process prediction/validation. Developed ab initio prediction models for the heat transfer coefficient (K_v_). The overall K_v_ is computed ab initio as the sum of the solid contact, gas conduction, and radiative heat transfer components

The quasi-steady one dimensional (1D) heat and mass transfer model described in the “Generation and Use of Design Space” section is usually referred to as the “Lyo-Calculator” and is illustrated below in the top portion of Figure 8. There are two important ways of using the steady-state mathematical models or two models as illustrated in Figure 8(d–f):

1. If one knows K_v_ and R_p_, one can calculate the unknown outputs (forward use)⁠—the standard use of a Lyo-calculator (9).
2. If one does not know R_p_ (or K_v_ ) to begin with but has the experimental cycle output, then one can analyze the unknown R_p_ (or K_v_ ) by fitting (backward use) (19,27).

For a given collapse temperature, the predicted critical output process parameters can be confirmed with a minimal number of experiments as illustrated in Figure 8.

### Scale-up and Transfer

Another opportunity to use/apply modeling is during scale-up and tech transfer to manufacturing between lyophilizers. In the subsequent discussions, two lab-scale lyophilizers, named as LabLyo1 and LabLyo2, and two pilot-scale lyophilizers, PilotLyo1 and PilotLyo2, will be used as examples.

During scale-up and tech transfer to manufacturing, differences in heat transfer (K_v_) and mass transfer (including choke flow limit) and product resistance (R_p_) arising from differences in scale, design, and geometry of the freeze-dryer, product, and environment (T_n_, class 100/particle-free environment) of the dryer need to be considered.

#### Class 100/Particle-Free Environment (T_n_)

**Fig. 9:**
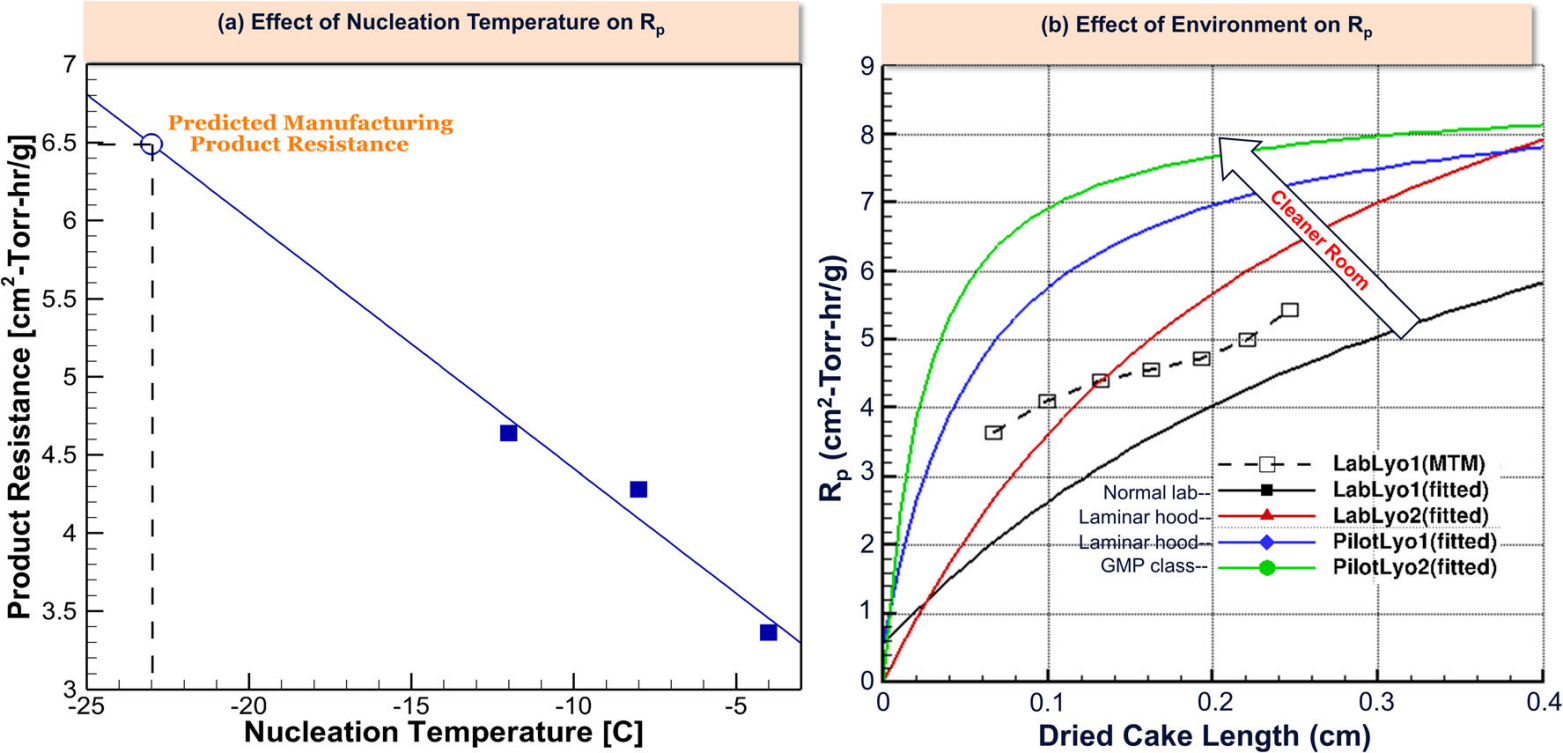
Effect of nucleation temperature and environment differences (particle-free/class 100 area) on mass transfer differences (R_p_). **a** Effect of nucleation temperature on R_p_. **b** Effect of environment on R_p_

A key factor that needs to be considered during transfer to manufacturing is the environment. The particle-free environment in manufacturing affects the nucleation temperature which affects the morphology of ice. This in turn affects the product resistance (R_p_), affecting the drying rate or mass transfer rate. Product resistance for an amorphous formulation product as a function of nucleation temperature was determined and plotted as illustrated below in Figure 9 a using the quasi steady-state model as described above. The R_p_ of the given formulation for a manufacturing environment where the nucleation temperature is typically approximately −23 °C was estimated from the curve. The specific surface area obtained from Brunauer-Emmett-Teller (BET) analysis can be further measured for each nucleation temperature case, which was found to linearly correlate with R_p_ by Rambhatla et al (28). As per Figure 9 b, it was found that for a given product run on different lyophilizers in different environments, the lower environment particle level in GMP conditions leads to lower ice nucleation temperature and therefore higher R_p_.

#### Equipment capability (minimum controllable chamber pressure as a function of sublimation rate)

**Fig. 10:**
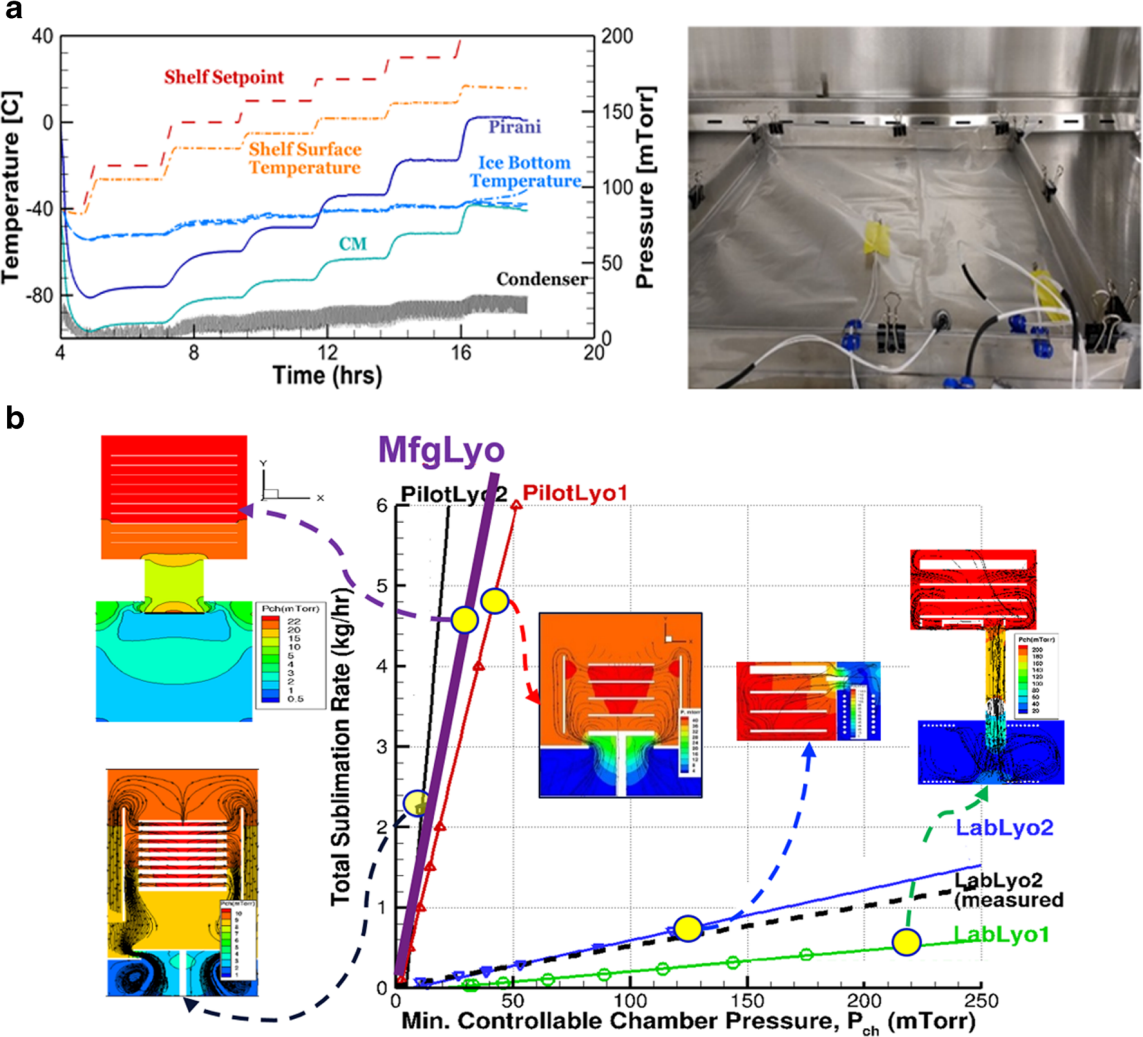
Equipment capabilities for various lyophilizers determined through ice slab experiments and CFD modeling**. a** Ice slab experiments and process data. **b** CFD modeled equipment capability and flow field of pressure

Typically, as part of the characterization of the freeze dryer, the equipment capability—the safety boundary without losing control of the chamber pressure—is assessed/determined through ice slab experiments (19) shown in Figure 10 a, which involve several experiments where the shelf temperature and chamber pressure are raised incrementally. The sublimation rate and the stable chamber pressure achieved are recorded and are plotted to determine the minimum controllable chamber pressure by the equipment at a given sublimation rate.

This is a cumbersome exercise and consumes significant time on the manufacturing line. Using computational fluid dynamics (CFD), the equipment capability can be easily estimated/predicted through simulations (19, 20) as illustrated below in Figure 10 b. Through CFD modeling, one also gains a deeper understanding of the pressure distribution and vapor flow speed in the entire equipment which helps in understanding process non-uniformity, condenser overloading, etc.

#### Heat Transfer Coefficient (K_v_)

**Fig. 11:**
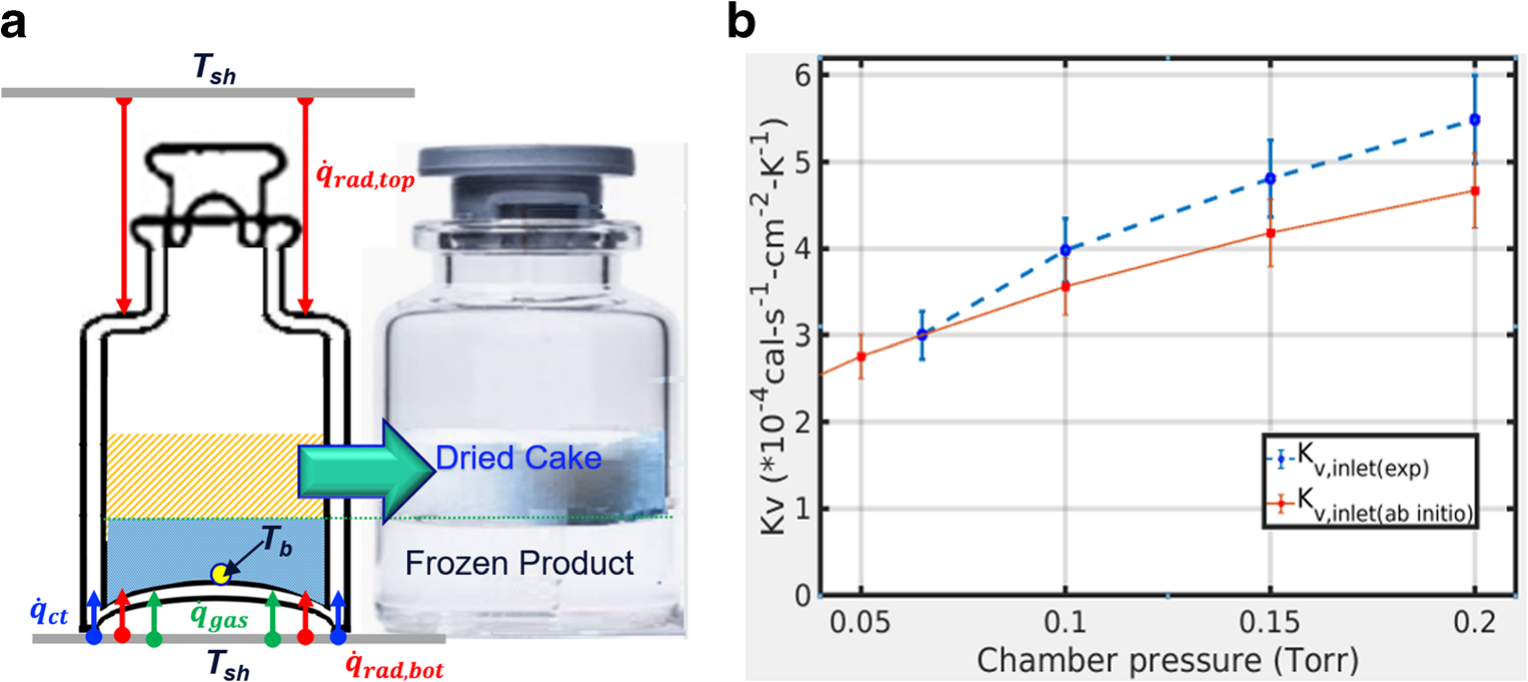
Developed ab initio prediction models for heat transfer coefficient (K_v_). The overall K_v_ is computed ab initio as the sum of the solid contact, gas conduction, and radiative heat transfer components ${\dot{q}}_{gas},{\dot{q}}_{rad}\ \mathrm{and}\ {\dot{q}}_{ct}\ \mathrm{are}\ \mathrm{the}\ \mathrm{heat}\ \mathrm{transfer}\ \mathrm{rate}\ \mathrm{through}\ \mathrm{gas}\ \mathrm{conduction},\mathrm{radiation}\ \mathrm{and}\ \mathrm{solid}\ \mathrm{contact},\mathrm{respectively}$ (9). **a** Three mechanisms of heat transfer to a vial. **b** Ab initio model vs. measured K_v_ for LabLyo2, 6R vial

A sublimation test with water runs is performed to determine the vial heat transfer coefficient (K_v_) for a given dryer. Since K_v_ is dependent on vial configuration and chamber pressure, every time either is changed, during the life cycle management of the product, sublimation tests need to be performed. Changes in K_v_ due to changes in vial configuration and chamber pressure can be relatively easy to predict with the use of an ab initio heat transfer model that, as illustrated in Figure 11 a, considers the conductive, radiative, and solid contact heat transfer contributions instead of running experiments on a manufacturing freeze dryer. For example, Figure 11 b shows that on a lab-scale lyophilizer, PilotLyo1, the modeled K_v_, is in acceptable agreement with the experimentally obtained K_v_ for PilotLyo1 (with the error estimated). It is seen from Figure 11 b as well as in Figure 3 earlier that experimental Kv for lyophilizers in typical operating pressure (50–250mTorr) ranges between 2 and 8 cal·s^−1^·cm^−2^·°C^−1^ (or between 4 and 11 Joule·h^−1^·cm^−2^·°C^−1^), and the modeled Kv would need to be carefully examined if it is found out of this range.

## Case Studies

### Construction of Design Space

**Fig. 12:**
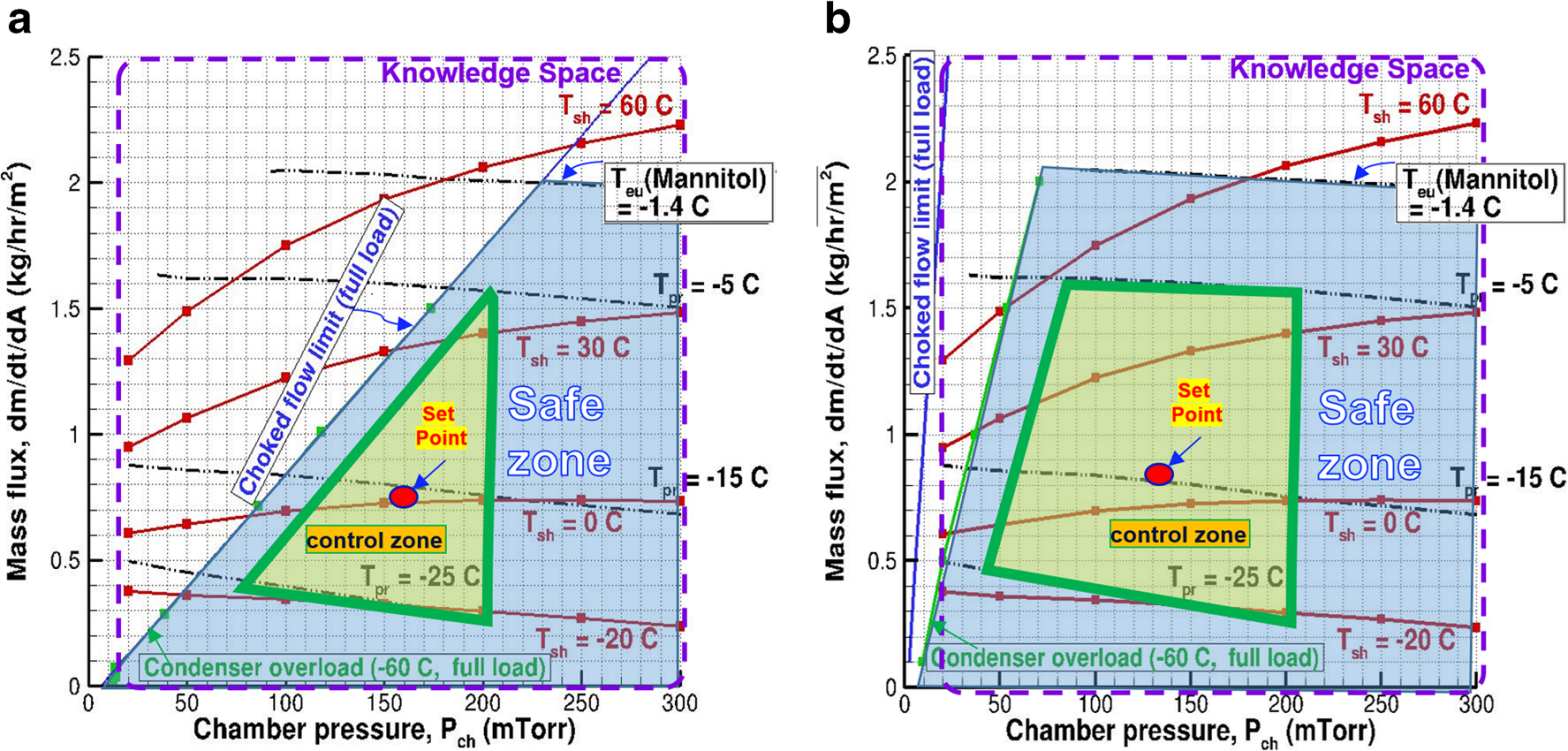
Design spaces created by coupled CFD and quasi-steady-state models to predict the equipment and process performance and guide operation: Knowledge space includes the range of inputs that are studied (inside of purple boundary); safe operating zone is bounded with choked flow limit and critical product temperature (blue region); control zone is bounded by the preferred range of maximum product temperature and chamber pressure (inside of thick green triangle or quadrilateral). The black traces are the calculated product temperature isotherms, where T_eu_ is the experimental critical product temperature. The red traces are the calculated shelf temperature isotherms. The equipment capability is represented by the solid blue line (due to choked flow) and also the thin green line (due to condenser overload). **a** For LabLyo2. **b** For PilotLyo1

Having characterized the freeze dryer and the manufacturing environment, the quasi steady-state model coupled with the CFD simulations can be used to construct a predictive knowledge space following the procedure described in the “Generation and Use of Design Space” section and can be employed to establish the equipment and process performance at the manufacturing scale (19). Figure 12 a and Figure 12 b, for example, show the design spaces computed based on the quasi-steady model for 5% mannitol in 6R vials on LabLyo2 and PilotLyo1, respectively. Within the design space/safe zone, a proven acceptable range, operating range/control space, and a set point can be created as illustrated in Figure 12a and b.

Construction of such a design space helps to identify the optimal conditions for a lyo process, the limits of failure, and the limits (ranges) for validation and the limits for process control for a given vial configuration, equipment, and manufacturing environment. Additionally, it can be used to predict the effect of variations in process conditions, on the process performance, and product quality attributes which helps in understanding the effects of excursions/deviations during manufacturing. Remember again that the validity and accuracy of the design spaces created on the manufacturing scale lyophilizer for the target product are completely dependent on the accuracy of the inputs to the model, including the scale-up strategy of K_v_ and R_p_ for the laboratory to manufacturing scale. The model can be further improved and validated along with more at-scale experimental data gathered as was discussed in the “Determination of Primary Drying conditions and Construction of Design Space” section.

### Effect of Batch Sizes (Product Load), Fill Volume, and Dose Strength

**Fig. 13:**
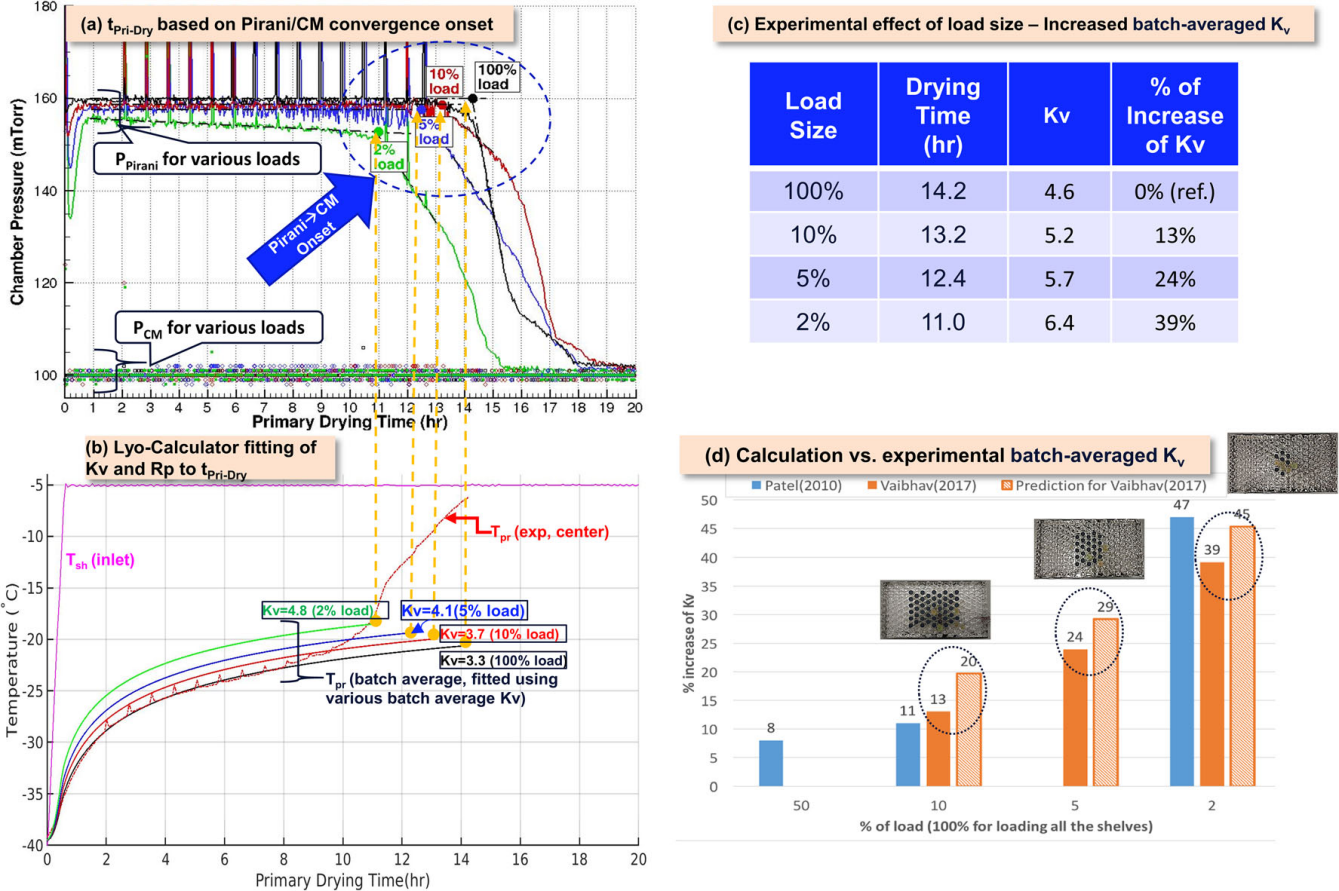
Effect of product load size differences on heat transfer, K_v_. **a** Determination of t_Pri-Dry_ based on Pirani/CM convergence onset, where the black, red, blue, and green solid lines are the Pirani pressure data for the 100%, 10%, 5%, and 2% loads of 5% Mannitol on LyoStar3, respectively. **b** Lyo-Calculator fitting of K_v_ and Rp to t_Pri-Dry_. The red dashed line with spikes is the measured center vial temperature for the 100% load case, whereas the solid black, red, blue, and green solid lines are the batch average temperature, fitted using using various Kv to t_Pri-Dry_ of the 100%, 10%, 5%, and 2% loads, respectively. **c**, **d** Experimental and predicted effect of load size in table and chart view, respectively. The percentage increase of K_v_ (batch-averaged by fitting to t_Pri-Dry_) relative to 100% load for 100%, 10%, 5%, and 2% partial loads on LyoStar II (in Patel et al. 2010, blue bar) and LyoStar 3 (experiment in dark orange bar and prediction in light orange bar), respectively

Product load or batch size influences the process performance, especially the primary drying time and heat transfer coefficient, and regulatory agencies expect revalidation of the process when the batch size is changed from within the validated range. For example, partial load drying process were performed on LabLyo1 with 100%, 10%, 5%, and 2% loads, and the associated heat transfer coefficient, K_v_, changes across load sizes were studied using first principles heat transfer model mentioned in earlier discussions. In Figure 13 d, the modeled change in batch averaged K_v_ was compared with those experimentally extracted (29) (using quasi-steady state model as shown in Figure 13a–c) and reached good agreement (5~7% difference).

**Fig. 14:**
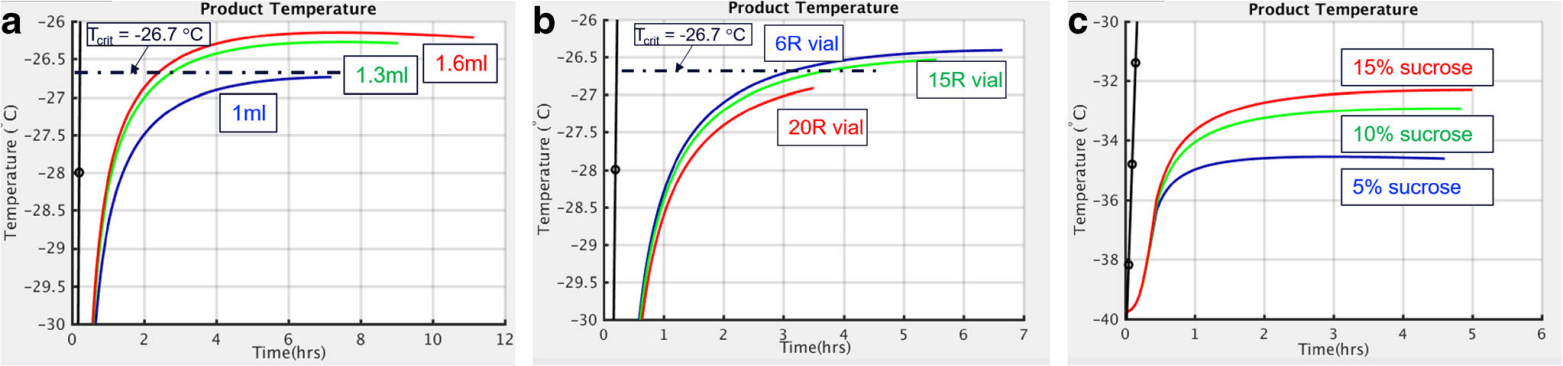
Effect of **a** fill volume, **b** vial size, and **c** solid content concentration/R_p_ on T_pr_(t) and t_PriDry_. In each subfigure, the red, green, and blue curves are the predicted product temperature in primary drying for each of the three varied conditions. The black dashed dot lines are the critical product temperature

Similarly, regulatory agencies expect revalidation of the process when the fill volume and/or dose strength is changed as they influence the lyophilization process performance and, consequently, the product quality attributes. Mathematical models based on quasi-steady-state, as described above, can be used to evaluate and predict the impact of changes in the vial sizes, fill volume, and dose strength on the process performance (product temperature) as illustrated below in Figure 14. Figure 14 a shows the modeled impact of changes in the fill volume on an amorphous-based formulation with 12% solid on a lab-scale lyophilizer, and Figure 14 b shows the impact of changing vial sizes for 1 mL of the same formulation. Figure 14 c shows the modeled impact of the solid content of sucrose.

The results of simulations/predictions can be used to assess whether the impact of those changes is significant, insignificant, or within the acceptable criteria and to decide the need to revalidate the process or not. Similar to the design spaces presented in the “Construction of Design Space” section, again the validity and accuracy of the predicted influence of the dosage form selection on the lyophilization process performance are completely dependent on the accuracy of the inputs to the model. The properties of the final formulated product such as Tc/Teu are critical and are a function of nature of API and corresponding dose strength. In case of mAbs, for example, it is well documented that the delta between Tc and Tg’ increases as a function of increasing protein concentration thereby allowing drying operation to occur at higher temperature. Similarly, increasing fill volume (or correspondingly lowering vial dimensions) imposes greater vapor flow restrictions during the drying process as illustrated in Figure 14 a and b. However, increasing concentration also results in an increase in mass transfer resistance (Figure 14 c) and thus caution must be taken to optimize the cycle for uniform drying without causing cake collapse. Additionally, maximum batch size should be established by taking not only the equipment capability such as minimum controllable chamber pressure as a function of sublimation rate but also by characterizing critical process parameters and their corresponding impact on product quality attributes as documented in Patel et al. (21) and Kshirsagar et al. (23).

### Controlled Ice Nucleation Technology and Its Effect on Product Resistance

All solutions undergo supercooling during the freezing step. Supercooling occurs when nucleation of ice occurs at solution temperatures well below the equilibrium freezing point for the formulation. Conventional lyophilizers cool solutions by decreasing the shelf temperature over a specified time. Ice nucleation is not controlled during this approach and occurs randomly over a wide range of solution temperatures. There is often a higher degree of supercooling in the aseptic manufacturing area compared with the preparation of samples in a laboratory environment. Controlling the temperature at which ice nucleates can drastically reduce the variability between the vials on a shelf and between shelves, both at small scale and at full scale. Reducing the variability can ensure all product in all vials dry at a similar rate and should exhibit similar quality attributes such as appearance residual moisture and reconstitution time. This can have an added advantage of significantly reducing primary drying time. The possibilities for reducing variability and lyophilization processing time have increased the interest of pharmaceutical companies in CIN.

Controlled ice nucleation is a recent technology used during the freezing step of lyophilization that can reduce inter-vial variability in ice nucleation temperature. Reducing the variability in ice nucleation temperature can reduce the differences in product resistance, R_p_, during drying so that all vials in the batch exhibit similar behavior. Besides, a reduction in product resistance can reduce the drying time especially when nucleation occurs at higher temperatures. A reduction in R_p_ occurs when there is a decrease in the surface area of ice as a result of conducting CIN at warmer temperatures resulting in large ice crystals that leave behind large pores in the drying solid. This can additionally reduce the interfacial interactions for molecules that are sensitive to interactions at the ice interface (30). The larger pores resulting from CIN may improve reconstitution time for highly concentrated formulations and formulations containing large molecules by making it easier for the diluent to penetrate the lyophilized solid (31). Improvements in the appearance of lyophilized solids may also be a result (32, 33).

Multiple methods have been investigated for controlling the nucleation of ice (34). Two CIN methods are available at full scale and one is available at a laboratory scale. The methods available at the laboratory and full scale include rapid depressurization using ControLyo® and the introduction of an ice fog using VERISEQ® nucleation. FreezeBooster® also uses an ice fog for seeding nucleation and is available at a laboratory scale.

A recent survey conducted by LyoHub found that more than 10 pharmaceutical companies are testing and/or implementing rapid depressurization CIN technology at scale, for multiple modalities, including monoclonal antibodies, vaccines, and gene/cell therapy products. A similar number (more than 6–10) of pharmaceutical companies are testing ice fog technology at scale, for monoclonal antibodies, vaccines, and small molecules. Both technologies are amenable to implementation on new lyophilizers as well as to retrofitting current lyophilizers. In either case, depending on the CIN technology, modifications to the equipment design are needed. For example, in ControLyo® technology, additional depressurization valve(s) may be installed on the lyophilization chamber. Further, the depressurization valves need to be provided with additional nozzles to meet “Clean in Place” requirements. CIN software may be integrated into the lyophilizer control system or may be executed via a separate control system, and the output CIN parameters may be evaluated either as part of the lyophilizer batch record or separately using a validated system output when controlling CIN operation with a separate control system.

The typical goals for implementing CIN are to reduce variability and to reduce lyophilization processing time. The reduction in processing time may be more substantial for some formulations than for others. For example, amorphous formulations with low critical product temperatures often require conservative processing conditions to prevent collapse during primary drying. The conservative conditions often lead to longer than desired processing times. The use of controlled nucleation for such formulations can drastically reduce processing time. Experiments conducted at Baxter Healthcare using a 5% sucrose solution suggest that the processing time can be reduced by as much as 25 h at a laboratory scale (Table I).

**Table I:** Comparison of lyophilization cycle conditions for 5% sucrose 5 mL in a 10 mL vial

Technique	Primary drying shelf temperature (°C)	Primary drying chamber pressure (mTorr)	Product temperature (°C)	Total cycle time (hours)
Conventional ice nucleation	-25	100	-33	95
Controlled ice nucleation at −5 °C	-21	100	-34	70

Several factors should be considered/studied at a laboratory scale when designing a process using CIN (Table II). One consideration is that the nucleation of ice starts at the top of the formulation and proceeds downward. This is the opposite of what occurs when using conventional (random) nucleation.

**Table II:** Considerations when using controlled ice nucleation

Factor to consider	Comments
For rapid depressurization	
Gas type: N_2_ or Ar	Argon may be necessary when using vials 6 mL or less in size.
Depressurization	Rate and magnitude of depressurization.
For ice fog	
Dispersion of ice nuclei	Adequate dispersion of the ice fog throughout the entire product chamber.
For both methods	
Ice nucleation temperature	Solution temperature prior to CIN affects success of CIN and also the drying time.
Hold time prior to CIN	Ensure sufficient time to equilibrate solution temperature in all vials to ensure success of CIN.
Ramp rate post ice nucleation	Slow ramp may encourage additional ice crystal growth.

The factors described in Table II should be considered when designing studies at a laboratory scale. The time needed for equilibration of temperature before ice nucleation may differ for different fill volumes and vial sizes. Insufficient equilibration time can prevent nucleation from occurring when desired. Ramp rates post-nucleation should also be considered. However, this is typically dictated by the capability of the lyophilizer at full scale. Most lyophilizers cannot proceed any faster than about 1 °C/min at full scale.

Transferring the process to full scale relies on typical lyophilizer qualification as described in other sections of this document. One of the important variables at full scale includes proving adequate control of shelf temperature under various load conditions. Increasing the thermal load in the lyophilizer may require longer hold times before ice nucleation to ensure equilibration of solution temperature in the vials.

Once the suitable CIN technology is selected, extensive characterization of the CIN process should be initiated. As a general strategy, small-scale CIN experiments may be first performed to determine the minimum (worst case) ice nucleation conditions for successful CIN. Additional experiments may be performed to evaluate the impact of selected CIN parameters on lyophilized cake attributes such as residual moisture. This will help establish the boundary conditions for the CIN process parameters to achieve the desired process/product performance. Once the small-scale ranges are defined, CIN cycles may be performed at the manufacturing scale to establish the CIN parameter robustness at scale.

Proving the method is operational post-installation at the manufacturing scale may require engineering batches that examine the performance of the CIN method apart from examining the performance of other usually tested lyophilization parameters. For example, rapid depressurization requires that the gas leaves the chamber as fast as possible. This may be hindered by the size of the port, the actuation valve, or if a sterilizing grade filter is used on the exit of the port. If so, additional ports may be necessary. Appropriate use of the ice-fog method requires that the ice nuclei rapidly flow into the product chamber and reach all vials located on all shelves. Therefore, it is important to consider the flow patterns of the ice nuclei in the chamber.

Hold times and cooling ramp rates may be important in decreasing the variability of ice nucleation and crystal growth. Some studies suggest that some molecules may be sensitive to long residence times in the freeze concentrate above the glass transition (T_g_’) and may adversely impact stability. Results from Merck labs showed that for certain proteins and viruses, longer time in solution (TIS) during the CIN shelf temperature may lead to degradation during the frozen (35). This would necessitate limiting the pre-ice/post-ice nucleation hold time range, and might even render CIN an unfavorable option in some cases.

Apart from the CIN parameter optimization/robustness, media fills also need to be performed to ensure that the contents of the lyophilizer will remain sterile post CIN. CIN conditions should be simulated (or even challenged) during the media fills. For example, during ControLyo®, media fill studies may be performed to evaluate the ability of the fill-finish process (including CIN) to maintain sterility, using worst-case CIN parameters (e.g., highest depressurization magnitude) as the worst case for sterility. The impact of high pressure on microbial growth may also need to be evaluated before performing the media fills.

## Summary

The validation activities of pharmaceutical lyophilization for stage 1 (process design), stage 2 (process qualification), and stage 3 (continued process verification) are considered in this work along with relevant case studies. In part I, the process design approach relying on generating a design space for a given product and equipment combination is presented and illustrated with examples from practice. Applications of modeling in process design and scale-up are also presented while showcasing the impact of facility, equipment, and K_v_. New and upcoming approaches to process improvement product monitoring, and process understanding with an emphasis on CMC requirements are discussed as well. Furthermore, illustrative case studies are documented for multiple vial sizes, fill volumes, and dosage strengths to demonstrate the value of modeling. These activities are aimed at enhancing process understanding in preparation for stages 2 and 3 of the validation processes described in the companion part II of the paper.
